# Microstructure Formation and Mechanical Properties of Metastable Titanium-Based Gradient Coating Fabricated via Intense Pulse Ion Beam Melt Mixing

**DOI:** 10.3390/ma16083028

**Published:** 2023-04-11

**Authors:** Mofei Xu, Xiang Yu, Shijian Zhang, Sha Yan, Vladislav Tarbokov, Gennady Remnev, Xiaoyun Le

**Affiliations:** 1 School of Physics, Beihang University, Beijing 100191, China; 2 Beijing Advanced Innovation Center for Big Data-Based Precision Medicine, School of Medicine and Engneering, Beihang University, Beijing 100191, China; 3 Beijing Key Laboratory of Advanced Nuclear Energy Materials and Physics, Beihang University, Beijing 100191, China; 4 Institute of Heavy Ion Physics, Peking University, Beijing 100871, China; 5 School of Advanced Manufacturing Technologies, National Research Tomsk Polytechnic University, 634050 Tomsk, Russia

**Keywords:** Ti-Cr, alloy coating, intense pulse ion beam melt mixing, compositionally gradient, surface morphology, phase structure, mechanical properties

## Abstract

The unique flash heating characteristics of intense pulsed ion beams (IPIB) offer potential advantages to fabricate high-performance coatings with non-equilibrium structures. In this study, titanium-chromium (Ti-Cr) alloy coatings are prepared through magnetron sputtering and successive IPIB irradiation, and the feasibility of IPIB melt mixing (IPIBMM) for a film-substrate system is verified via finite elements analysis. The experimental results reveal that the melting depth is 1.15 μm under IPIB irradiation, which is in close agreement with the calculation value (1.18 μm). The film and substrate form a Ti-Cr alloy coating by IPIBMM. The coating has a continuous gradient composition distribution, metallurgically bonding on the Ti substrate via IPIBMM. Increasing the IPIB pulse number leads to more complete element mixing and the elimination of surface cracks and craters. Additionally, the IPIB irradiation induces the formation of supersaturated solid solutions, lattice transition, and preferred orientation change, contributing to an increase in hardness and a decrease in elastic modulus with continuous irradiation. Notably, the coating treated with 20 pulses demonstrates a remarkable hardness (4.8 GPa), more than twice that of pure Ti, and a lower elastic modulus (100.3 GPa), 20% less than that of pure Ti. The analysis of the load-displacement curves and *H*-*E* ratios indicates that the Ti-Cr alloy coated samples exhibit better plasticity and wear resistance compared to pure Ti. Specifically, the coating formed after 20 pulses exhibits exceptional wear resistance, as demonstrated by its *H*^3^/*E*^2^ value being 14 times higher than that of pure Ti. This development provides an efficient and eco-friendly method for designing robust-adhesion coatings with specific structures, which can be extended to various bi- or multi-element material systems.

## 1. Introduction

Due to its advantageous properties, including excellent biocompatibility, superior hydrogen storage capacity, high strength-to-weight ratio, and low cost, titanium (Ti) is a highly sought-after material and has been widely used in biomedicine, aerospace, and hydrogen storage [1,2,3,4,5]. Previous studies [6,7,8] have shown that alloying Ti with various elements can enhance its strength, wear, and corrosion resistance. Notably, the addition of Chromium (Cr) into Ti has been reported to promote the formation of metastable and Laves phases, thereby improving the material’s service performance [9,10,11,12,13,14]. Fabricating Ti-Cr alloy coating, rather than manufacturing bulk Ti-Cr alloy, has emerged as a more efficient and cost-effective method [10,15,16,17,18,19], which not only improves surface performance, but also preserves the merits of the substrate material.

In recent decades, intense pulsed ion beam (IPIB) technology has been successfully applied in material surface treatment for its flash heating effect [20,21,22,23,24]. IPIB is characterized by short pulse duration (within 1 μs), short range (~μm scale), and high power density (up to 10^11^ GW/m^2^) [25,26,27]. Upon interaction with the material surface, ions deposit energy in a shallow layer, leading to a rapid surface heating rate of up to 10^11^ K/s. The surface layer subsequently melts within tens to hundreds of nanoseconds and rapidly cools down at a rate of 10^9^ K/s [25,28]. Considering these characteristics, intense pulsed ion beam melt mixing (IPIBMM) has merged as a promising technology for surface alloying. Specifically, for a substrate pre-coated with a film, IPIB irradiation induces the surface temperature rise, resulting in the melting of the film and a certain depth of the substrate. Through diffusion and convection effects, the film and substrate can be mixed to form an alloy coating.

In comparison to conventional methods, such as physical vapor deposition [29,30], electroplating [31], and thermal spraying [32,33], the alloy coating prepared by IPIBMM offers the potential for metallurgical bonding with the substrate. The coating is challenging to detach during application due to its stronger adhesion to the substrate. Moreover, the mutual diffusion of the film and substrate creates an opportunity to form the composition gradient coating with a continuous variation in thermal and mechanical properties. This property is beneficial for eliminating the interlaminar stress caused by the discrepancy of the material parameters. Additionally, non-equilibrium processes on the material surface induced by the transient thermal shock of IPIB can change the microstructure of the material surface layer [34,35,36,37], providing the possibility to optimize the coating performance. In previous studies, it has been found that the film-substrate mixing can occur through IPIB irradiation for single-layered Cu/Mo, Pb/Fe systems and multilayered Pb/Fe/Pb, Al/Cu/Fe, Al/Pb/Fe systems [38]. However, the obvious differences in thermodynamic properties between the film and the substrate may be unfavorable for the melt mixing process of the systems [39]. The IPIBMM method has been used for the preparation of Al-Ti alloy coating, and it has shown to improve corrosion resistance compared with the use of untreated materials [40].

Previous studies have demonstrated that IPIBMM is a viable surface alloying approach for some material systems. However, the evolution of the microstructure during irradiation proceeding and its effect on the mechanical properties of the coatings have not been systematically investigated. Additionally, the Ti-Cr alloy coating preparation by the IPIBMM method has not been explored. In this study, we calculated the material surface thermal field under IPIB irradiation, which demonstrates the melting process of Cr film-Ti substrate and verifies the feasibility of the IPIBMM method for this material system. We then adopted a combined strategy of magnetron sputtering and IPIB irradiation to prepare the Ti-Cr alloy coating in order to produce metastable and composition gradient structures. We thoroughly researched the element distribution, surface morphology, and phase composition of the coatings treated by different IPIB pulses to better comprehend the microstructure evolution and formation mechanisms of the coatings, which is critical to the effective utilization of the IPIBMM method. Finally, we analyzed the mechanical properties of the Ti-Cr alloy coating samples and explored the relationship between structural changes and improvement in properties. Our work sheds new light on the fabrication of high-performance Ti-based alloy coating and provides a valuable reference for designing coatings for various material systems.

## 2. Materials and Methods

**Figure 1:**
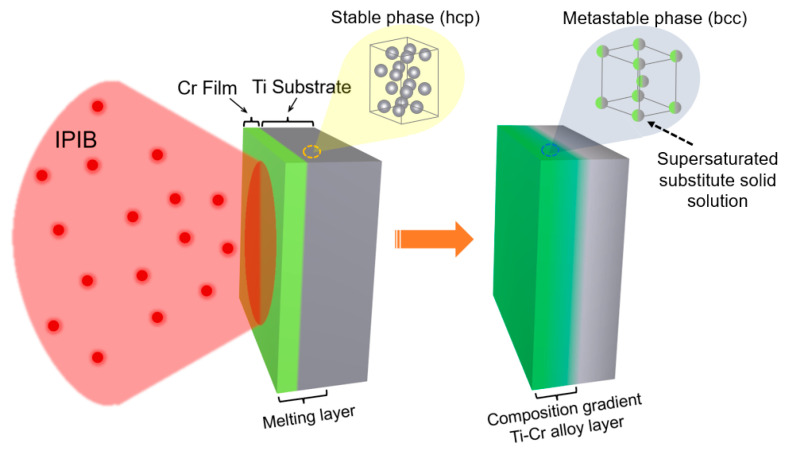
Scheme of the preparation process of Ti-Cr alloy coating through IPIBMM.

In this research, the surface alloy coating was prepared by magnetron sputtering and subsequent IPIB irradiation. High-purity (99.995%) Ti sheets procured from Trillion Metals Co., Ltd. (Beijng, China), were cut into small pieces with the dimension of 10 mm × 10 mm × 2.5 mm using the electron discharge machining method. All the obtained Ti substrates were polished by mechanical grinding with silicon carbide grinding paper and then polished with a diamond suspension. After that, the Ti substrates were ultrasonically cleaned in alcohol and high-purity deionized water prior to coating. The Cr films were deposited onto four Ti substrates by magnetron sputtering. The sputtering process was carried out in a working chamber that was first evacuated to a base pressure of less than 6 × 10^−4^ Pa, and then argon gas was introduced in to maintain a total pressure of 0.49 Pa. The growth rate of the film was ~24 nm/min under the sputtering power of 45 W. The thickness of the Cr films was determined to be ~400 nm by cross-section observation using a scanning electron microscope, and the thickness was uniform for each sample. Subsequently, the samples coated with Cr film underwent IPIB irradiated at room temperature to form alloy layers. The preparation process of alloy layers using the IPIBMM method is illustrated in Figure 1. The IPIB irradiation was carried out by TEMP-4M accelerator with a magnetically self-insulated diode at Tomsk Polytechnic University. This apparatus is known to generate an ion beam of 70% C^n+^ and 30% H^+^, with a current density ranging from tens to hundreds of A/cm^2^ [41,42,43,44]. In this work, an accelerating voltage of 190 kV and a pulse duration of 100 ns were employed. To facilitate clarity in presentation, the samples utilized in this study have been labeled as follows: the polished pure Ti sample has been designated as the “original sample”, the pure Ti substrate coated with Cr film has been referred to as the “unirradiated sample”, and the “irradiated samples” were prepared by treating the unirradiated samples with IPIB at an energy density of 2 J/cm^2^ for 1, 5, and 20 pulses, respectively.

The finite element method (FEM) software Comsol Multiphysics 6.0 was employed to compute the temperature field distribution in the irradiated sample during IPIB irradiation. The morphology of the surface and cross-section were analyzed by scanning electron microscopy (SEM) using a ThermoFisher (Waltham, MA, USA) Helios G4 CX DualBeam microscope in the secondary electron mode, where the electron accelerating voltage and current were set to 10 kV and 300 pA, respectively. To examine the cross-section, the samples were sliced electrically along the centerline. The resultant high temperature led to the formation of an oxide layer on the cross-section. This oxide layer was eliminated through the use of sandpapers with varying grain sizes. The polished cross-section was immersed in an etched solution (2 wt% HF, 10 wt% HNO_3_, 8 wt% H_2_O) for 10 s to enhance the SEM investigations. The element distribution analysis of the surface and cross-section were conducted by energy dispersion X-ray spectroscopy (EDS) using a Bruker (Billerica, MA, USA) X-ray detector at an accelerating voltage of 15 kV. The phase identification of the surface alloyed layers was investigated by X-ray diffraction (XRD) using Bruker D8 ADVANCE equipped with a high-resolution goniometer, a sealed tube X-ray source, and a scintillation detector. XRD utilized Cu-Kα radiation with a wavelength of 1.5406 Å; and a 0.05° scan step and a 1 s counting time were selected for analysis. Nanoindentation tests was performed by means of a STEP500 device equipped with a triangular pyramid diamond indenter, and a maximum load of 10 mN was selected for the tests. The error bars in the results originated from the calculation according to five tests on each sample. 

## 3. Results and Discussion

### 3.1. Simulation Results of Surface Layer Temperature Field

The temperature field distribution in the Ti substrate with the 400 nm Cr film induced by IPIB irradiation with energy density of 2 J/cm^2^ was calculated by FEM. The heat transfer process can be expressed by the Fourier thermal conduction equation:

(1) $$\rho \left(T\right)C\left(T\right)\frac{\partial T\left(z,t\right)}{\partial t}=\lambda \left(T\right)\frac{\partial^{2}T\left(z,t\right)}{\partial z^{2}}+P,$$

Here *ρ*, *C*, and *λ* are the density, specific heat, and thermal conductivity, respectively. The source term *P* is defined as:

(2) $$P=k\cdot d\left(z\right)\cdot f(t),$$

where *k* is the energy density, *d*(*z*) is the depth-normalized function of ion energy loss calculated by SRIM, and *f*(*t*) is the time-normalized function of current density distribution obtained experimentally using a Faraday cup. The initial condition is set to *T*(*z*,0) = 293.15 K, and the latent heats of Ti and Cr are considered in the calculation. 

**Figure 2:**
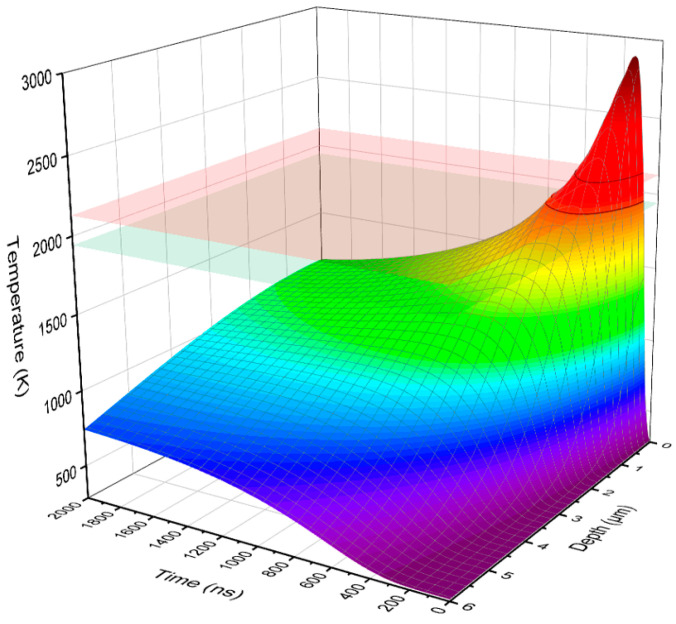
Spatiotemporal evolutions of temperature of the Ti substrate with 400 nm Cr film induced by IPIB irradiation, with an energy density of 2 J/cm^2^.

The spatiotemporal evolutions of temperature induced by IPIB irradiation on a Ti substrate with a 400 nm Cr film is shown in Figure 2. The simulation results indicated that the surface temperature of the sample rapidly increased to 2886 K within ~150 ns upon the impact of IPIB at 2 J/cm^2^, then decreased due to the heat transfer to the deeper regions. 

**Figure 3:**
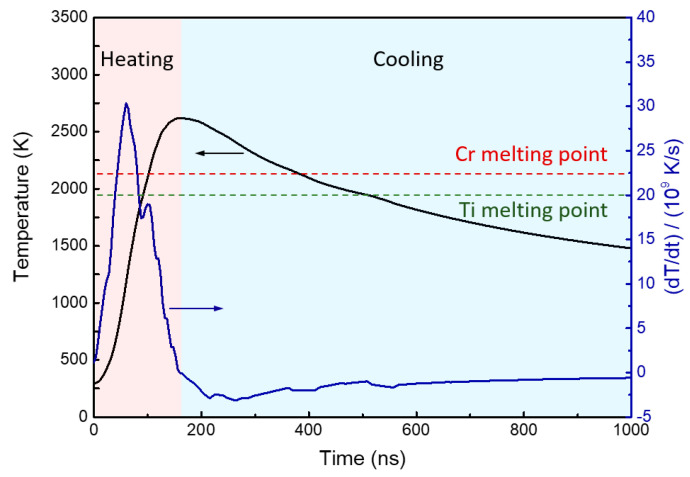
Variations in temperature and temperature change rate with time at a depth of 400 nm (interface between film and substrate).

**Figure 4:**
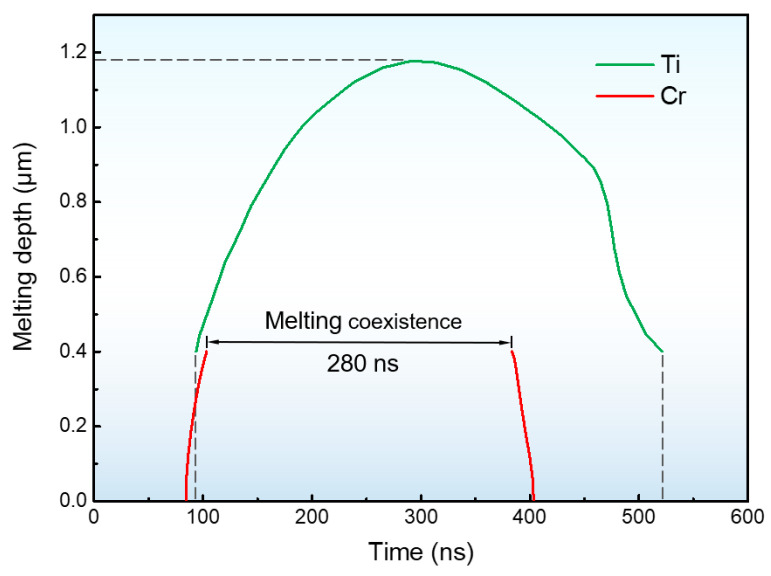
Evolutions of melting depth in the Ti substrate with 400 nm Cr film under IPIB irradiation.

Further analysis of the variations in temperature and its change rate with time at a depth of 400 nm (i.e., the interface between film and substrate) was performed, and the results are presented in Figure 3. The findings revealed that the highest heating rate and cooling rate at this depth could reach the order of 10^10^ K/s and 10^9^ K/s, respectively, which creates a non-equilibrium condition for the emergence of a metastable structure in the surface layer. Besides, the maximum temperature at the interface exceeded the melting point of Ti and Cr, indicating the occurrence of a melting coexistence period of the film and substrate. Figure 4 shows that the melting coexistence period of Ti and Cr lasted for 280 ns for 1 IPIB pulse. Moreover, the melting and solidification of Cr on the surface occurred prior to those of Ti on the subsurface. The existence of melt on the (sub)surface began at 86 ns and lasted until 520 ns. The maximum melting depth was estimated to be 1.18 μm. The presence of melt in the surface layer can facilitate effective homogenization of the composition inside the melt, and the mass exchange between the film and substrate can be more readily achieved, especially during the melting coexistence period.

### 3.2. Elements Distribution in Depth

**Figure 5:**
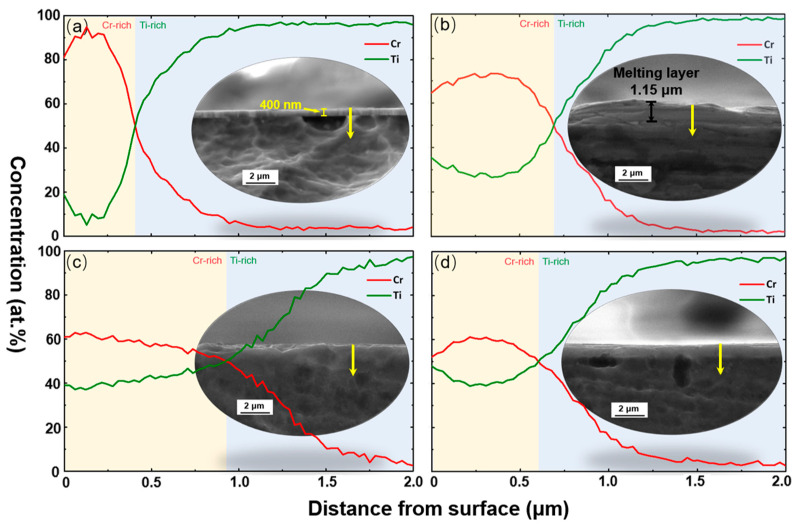
Cross-sectional EDS linear analysis of element distribution of Cr/Ti, according to the yellow arrow, for the (**a**) unirradiated sample, and (**b**–**d**) the irradiated samples with 1, 5, and 20 pulses of IPIB treatment, respectively.

Figure 5 presents the cross-section morphology and elements distribution of unirradiated and irradiated samples. The EDS data were obtained in the direction of the yellow arrow. The unirradiated sample displayed minimal mutual elements diffusion between the film and substrate during the film deposition (Figure 5a). As we used a substrate temperature of 400 °C during magnetron sputtering to improve film-substrate bonding, atomic diffusion was promoted between the film and substrate elements, resulting in the observed Cr concentration in the substrate region. Under IPIB irradiation, surface melting occurred, and the thickness of the melting layer was found to be ~1.15 μm (Figure 5b), which was in close agreement with the calculation value (1.18 μm). This led to a blurred interface between the film and the substrate due to surface layer remelting. Consequently, metallurgical bonding of the film and the substrate was achieved, evidently enhancing the coating’s adhesion to the matrix. 

The melt mixing of the film and substrate was activated under IPIB impact. In the molten state, the diffusion coefficient is significantly greater than that of the solid state. Furthermore, hydrodynamic instabilities resulting from thermal shock waves, as well as thermocapillary convection, facilitated the mass transport process between the film and the substrate [45,46]. This resulted in a decrease in the Cr concentration near surface region, as it migrated to the deep layers after IPIB treatment (Figure 5b–d). Correspondingly, the Ti concentration near the surface gradually increased and approached the concentration of Cr after 20 pulses. It is evident that the composition of the alloy layer formed by IPIBMM exhibited a continuous gradient distribution in depth.

Figure 5 illustrates the content balance depth, which refers to the depth at which the concentration curves of Ti and Cr intersect. For the unirradiated sample, the content balance depth was ~400 nm (Figure 5a). However, this depth increased to 685 nm and 930 nm after 1 and 5 pulses, respectively (Figure 5b,c), suggesting a continuous mass exchange between the film and the substrate during IPIB irradiation. Notably, the content balance depth dropped with 20 pulses of treatment (Figure 5d), which is likely due to surface ablation induced by IPIB impact [47,48].

**Figure 6:**
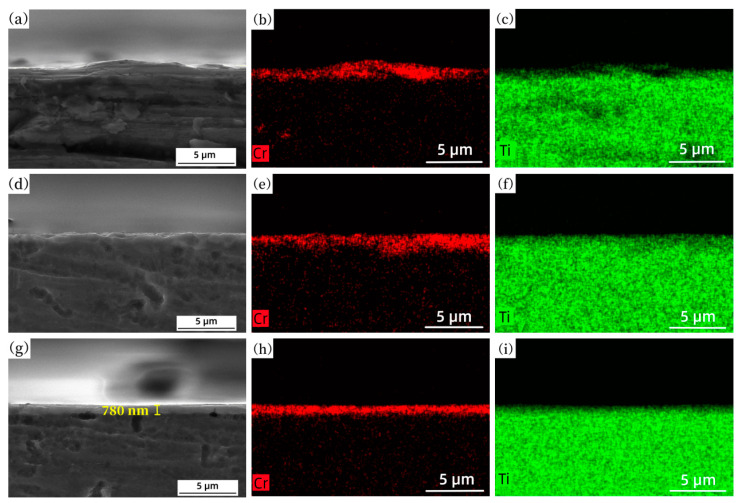
Cross-section EDS mapping of Ti-Cr alloy coating formed by (**a**–**c**) 1 pulse, (**d**–**f**) 5 pulses, (**g**–**i**) 20 pulses of IPIB treatment.

The cross-section of the Ti-Cr alloy coating formed by different IPIB pulses and the corresponding element distribution of Cr and Ti are shown is Figure 6. The alloy layer displayed uneven element distribution after 1 pulse treatment, with numerous Cr-rich areas and Ti-rich areas present (Figure 6a–c). The surface showed a large fluctuation and significant roughness (Figure 6a), which might be attributed to the ununiform composition distribution in the surface layer, intensifying the instability of fluid flow under the thermal shock of IPIB. However, subsequent IPIB irradiation promoted the redistribution of elements, resulting in a more uniform composition distribution and smoother surface of the alloy coating after 20 pulses (Figure 6g–i). The thickness of the alloy coating was ~780 nm. All the above results demonstrated that increasing the number of IPIB pulses was conducive to improving the uniformity of the mixing of film and substrate.

Based on the EDS mapping of the cross-section presented in Figure 6, it was found that the thickness of alloy coating obtained by 20 pulses was lower than that obtained by 1 or 5 pulses. This finding was consistent with the results obtained from the EDS line scan in Figure 5d. These phenomena provided strong evidence of surface ablation induced by IPIB. Previous studies by Yan et al. [39] confirmed the occurrence of ablation caused by IPIB, even at surface temperatures below the material boiling point. As the number of pulses increased, the element distribution became more uniform in the alloy coating, while the driving force for element migration to the deep layer was weakened. Consequently, further increases in the number of pulses led to the thinning of the alloy coating due to surface ablation. It is indicated that the number of IPIB pulses cannot be increased without limits, as it may cause complete depletion of the alloy layer.

### 3.3. Surface Morphology

**Figure 7:**
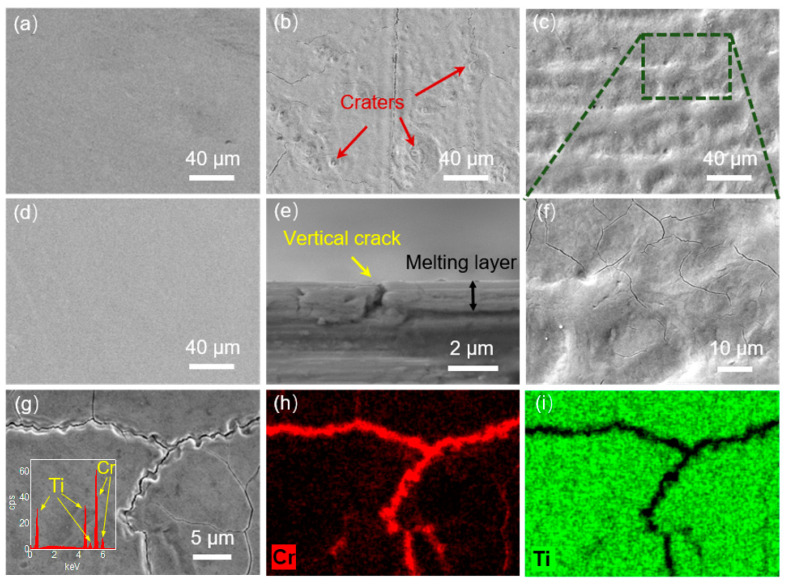
The surface SEM images of (**a**) unirradiated sample, (**b**–**d**) irradiated samples with 1, 5, and 20 pulses of IPIB treatment, respectively. (**e**) Cross sectional morphology of vertical crack on the irradiated sample. (**f**) Local enlarged view of the area marked by box in (**c**). (**g**–**i**) EDS mapping of the cracks on the irradiated sample surface.

Figure 7 exhibits the surface morphology of a 400 nm Cr film coated on Ti substrate before and after IPIB irradiation with different pulse numbers. It is observed that plenty of cracks and craters appeared on the surface after 1 pulse of IPIB treatment (Figure 7b). However, the surface defects decreased as the number of pulses increased. Cracks and craters were almost absent from the surface after 20 pulses (Figure 7d).

Upon irradiation, the surface displayed numerous craters, with their centers appearing in the form of a hole or facet, and some surrounded by an annular wavy structure (Figure 7b). Similar craters have been observed on other materials treated by IPIB [49,50]. The possible reasons for the crater formation have been previously discussed in the literature [51,52,53], including impurities, grain boundary thermal resistance, and vacancy gathering. The annular wavy morphology around the craters is thought to be formed by the recoil generated by a local eruption on the molten surface [54]. However, another possible reason in this study is the ejection of the subsurface melt. FEM calculation results indicated that the substrate melted first and crystallized later than the film at the interface. The volume expansion of a piece of molten metal surrounded by crystallized metal resulted in the ejection of the subsurface layer, which formed a crater on the surface. This effect weakened as the number of the pulses increased and the surface layer became more homogenized. Furthermore, the edges of the craters with a large curvature tended to migrate around due to the surface tension in the melting state [52]. As a result, the newly-formed craters were less frequent, and the sharp edges of previous craters became smoother after 5 pulses. The edges of the adjacent craters might interfere with each other during migration, leading to the formation of a complex wavy surface (Figure 7c). As the irradiation process continued, the surface tension redistributed the mass of the surface layer until a flat surface was obtained. After 20 pulses, it was challenging to detect any craters on the irradiated sample (Figure 7d).

The formation of surface cracking observed in the present study is probably attributable to the thermal expansion coefficient mismatch between Ti and Cr [55]. Under IPIB irradiation, the melted region of the surface layer rapidly cooled and solidified, creating tensile stress at the interface duo due to the different thermal expansion properties of the film and substrate. This stress caused cracks to generate at the interface and extend to the surface [56,57]. As shown in Figure 7b, plenty of large-size cracks were observed on the surface after 1 pulse irradiation. The cross-section morphology in Figure 7e clearly illustrates the vertical crack. Figure 7g–i exhibits the element distribution around the crack. Surface cracking exposed the substrate. This might provide the paths for Ti movement to the surface, thereby accelerating the progression of surface mixing. As irradiation continued, a uniform Ti-Cr mixing layer was formed on the surface. The composition gradient mixing layer replaced the two-layer system, significantly reducing the tensile stress caused by the local differences in the physical properties. This means that the cracking behavior was suppressed with further irradiation. Moreover, the surface melting induced by IPIB irradiation may have “cured” the pre-existing surface cracks. As depicted in Figure 7c, the large-size cracks disappeared after 5 pulses of irradiation, suggesting that they had been “cured”. However, some small cracks were still observed in the local enlarged view (Figure 7f), indicating the existence of localized uneven mixing areas on the surface after 5 pulses. Notably, there were almost no cracks on the surface after 20 pulses of irradiation (Figure 7d) due to the homogenization of the composition in the surface layer.

### 3.4. Phase Structure Analysis

**Figure 8:**
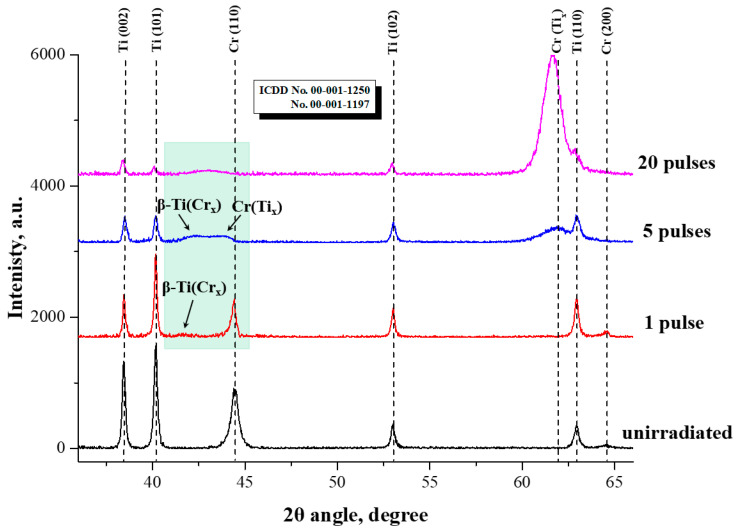
XRD patterns of Cr/Ti system samples before and after IPIB treatment.

The XRD patterns of the Cr/Ti system samples, before and after IPIB treatment, are presented in Figure 8. The unirradiated sample exhibited diffraction peaks of α-Ti with a hexagonal close-packed (hcp) structure and Cr with a body-centered cubic (bcc) structure, corresponding to the substrate and film, respectively. After 1 pulse of IPIB irradiation, a new diffraction peak appeared at a diffraction angle of ~41.5°, which is probably attributed to the formation of a β-Ti (Cr) supersaturated substitution solid solution. Similar findings have also been reported in the literature [19]. 

**Figure 9:**
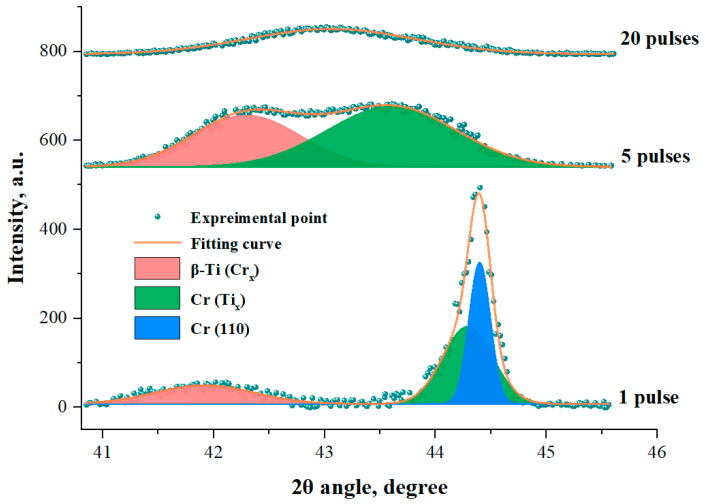
Local enlarged view of the XRD patterns of the irradiated samples.

In the Ti-Cr binary phase diagram, the eutectoid reaction occurs when the Cr content reaches ~12.5 at.%. However, IPIB irradiation enabled the attainment of much higher solid solubility than this value because it induced an ultra-high undercooling on the surface. The dissolution of Cr into the Ti lattice provided a stability effect for the metastable phase [58,59], which promoted the transformation of α-Ti into β-Ti. Moreover, the addition of Cr reduced the lattice parameters of β-Ti, due to the smaller atomic radius of Cr compared to Ti. Consequently, the diffraction peak of solid solution β-Ti (Cr) shifted towards the large angle side. Additionally, after 1 pulse of IPIB irradiation, the diffraction peak of Cr (110) became weak due to the partial mixing of Cr into the Ti lattice, whereas the dissolution of Ti into the Cr lattice also occurred. As a result, the diffraction peak of the solid solution Cr (Ti) was separated from the Cr (110) diffraction peak, resulting in the asymmetric shape of the Cr (110) diffraction peak. This phenomenon was observed more clearly from the local enlarged view in Figure 9.

As presented in Figure 8, the XRD patterns of the samples revealed distinct changes after multiple IPIB irradiation pulses. Specifically, a diffraction peak emerged at ~62° after 5 pulses, corresponding to the Cr (Ti) solid solution phase, which is probably attributed to the dissolution of Ti into the Cr (200) lattice. As irradiation continued, the diffraction peak of Cr (Ti) solid solution shifted left to 61.6° and became stronger. It is indicated that more Ti atoms entered into the Cr lattice. In contrast, the intensity of the Cr (110) diffraction peak gradually weakened with the increasing pulse number. This finding suggests that the preferred orientation of Cr transformed from (110) to (200) under IPIB irradiation.

The XRD pattern of the unirradiated sample revealed that the preferred orientation of the Cr deposition layer was the close-packed plane (110). However, with a higher surface energy, the secondary close-packed plane (200) had a higher growth rate than the plane (110) during crystallization. Additionally, the ultra-high undercooling induced by IPIB irradiation facilitated the retention of the Cr (200) crystal plane. Hence, the transformation of the preferred orientation of Cr from (110) to (200) occurred during the IPIBMM process.

Figure 9 presents a magnified view of the XRD patterns of the irradiated samples, focusing on the green region in Figure 8. The diffraction peaks of the supersaturated solid solutions β-Ti (Cr) and Cr (Ti) can be clearly distinguished. These two diffraction peaks showed large widths due to the concentration scattering phenomenon in the solid solution. The inhomogeneity of the composition in space, particularly within a depth of ~1 μm, covered by the detection range of XRD, resulted in a continuous gradient elements concentration. Therefore, the surface layer of the irradiated samples contained a series of solid solutions β-Ti (Cr_x_), with different Cr concentrations, and solid solutions Cr (Ti_x_), with different Ti concentrations. 

Compared with the sample irradiated with 1 pulse, the mixing of Ti and Cr in the surface layer of the sample irradiated with 5 pulses was more complete. The increased number of Cr atoms dissolving in the β-Ti lattice led to a decrease in the lattice parameter and a right shift of the β-Ti (Cr_x_) diffraction peak. Meanwhile, the increased number of Ti atoms in the Cr lattice induced a left shift of the Cr (Ti_x_) diffraction peak. The diffraction peaks of β-Ti (Cr_x_) and Cr (Ti_x_) gradually approached and merged into one peak after 20 pulses. It is demonstrated that IPIB irradiation promoted the mixing of surface elements, and the elements in the alloy coating were sufficiently mixed after 20 pulses.

### 3.5. Mechanical Properties

**Figure 10:**
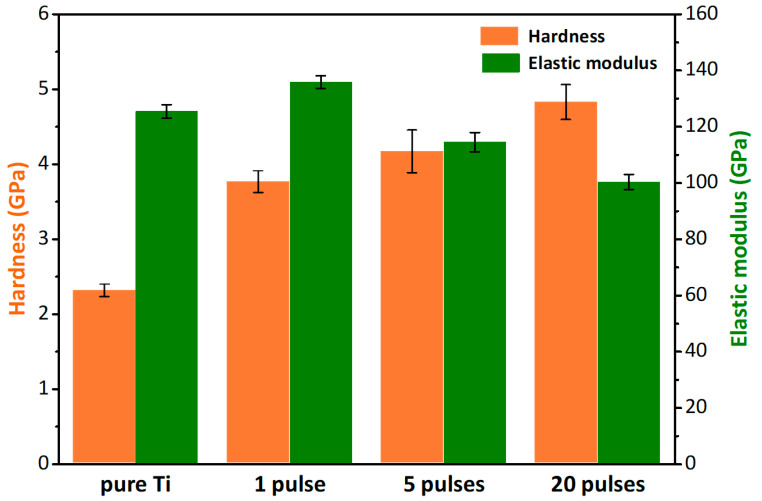
Hardness and elastic modulus of the pure Ti sample and the Ti-Cr alloy coating samples with different pulses irradiations.

The results of the nanoindentation tests on the pure Ti and Ti-Cr alloy coating samples are presented in Figure 10. The pure Ti sample showed a hardness of 2.3 GPa. After preparing the Ti-Cr alloy coatings on the pure Ti substrate using IPIBMM, the hardness of the sample surface was found to be significantly improved. Moreover, it is discovered that the hardness increased with an increase in the number of IPIB pulses. After 20 pulses, the hardness of the Ti-Cr alloy coating sample reached 4.8 GPa, more than twice that of pure Ti. This improvement in hardness was probably attributed to the solid solution hardening effect [18], which was further enhanced with an increase in solid solubility caused by irradiation. 

Interestingly, the elastic modulus of the Ti-Cr alloy coating sample treated with 1 IPIB pulse was slightly larger than that of pure Ti. With an increase in the number of pulses, however, the elastic modulus of the coating samples gradually decreased. After 20 pulses, the elastic modulus reduced to 100.3 GPa, about 20% lower than that of pure Ti (125.5 GPa). The elastic modulus is determined by the atomic bonding force, and this phenomenon could be explained by several reasons. Firstly, after the pure Ti substrate with Cr film underwent 1 pulse irradiation, the Cr atoms dissolved into the substrate. The addition of Cr diminished the crystal plane spacing of the surface layer and improved the atomic bonding force, resulting in an enhancement in the elastic modulus of the Ti-Cr coating sample after 1 pulse. Secondly, the further irradiation induced the preferred orientation and lattice transformation. The main arrangement plane of the Cr atoms changed from the close-packed plane (110) to the secondary close-packed plane (200). The lattice of Ti transformed from α-Ti (hcp) into β-Ti (bcc). Both of the changes resulted in a further atomic distance, thereby weakening the bonding force between atoms. Another reason for the decrease in the elastic modulus could be the decline in the content of Cr in the surface layer. Cr, with a smaller atomic radius, can play a role of reducing the lattice constant, but its content decreased due to ablation loss with an increase in the number of pulses.

**Figure 11:**
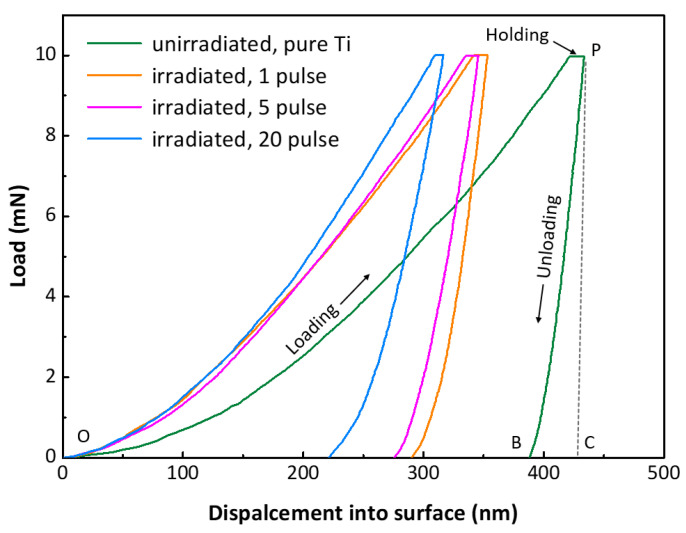
Load-displacement curves of the pure Ti sample and the Ti-Cr alloy coating samples with different pulses of irradiation.

Figure 11 exhibits the nanoindentation load-displacement curves of the surface layer of the pure Ti and Ti-Cr alloy coating samples. According to the literature [60,61], the elastic and plastic behavior of the surface layer can be analyzed from the load-displacement curves. The total deformation work *W*_t_ and plastic deformation work *W*_p_ were denoted by the area under the loading curve (*A*_OPC_) and the area enclosed by the loading and unloading curves (*A*_OPB_), respectively. The difference between *A*_OPC_ and *A*_OPB_, i.e., *A*_BCP_, represents the elastic recovery capability of the surface layer. The plastic factor *η*_p_, which can be used to assess the plastic deformation resistance of the surface layer, was determined by the ratio *W*_p_/*W*_t_. A lower value of *η*_p_ indicates a better resistance to plastic deformation. 

Table 1 displays the plastic factor *η*_p_ and the ratios of hardness and the elastic modulus of pure Ti and Ti-Cr alloy coating samples treated by different IPIB pulses. The pure Ti sample had a plastic factor *η*_p_ of 0.887, while the Ti-Cr alloy coatings showed a decrease in the value of *η*_p_ after the formation of the coatings on the Ti substrates. Further IPIB treatment on the irradiated samples caused a continuous decline in the value of *η*_p_. After 20 pulses, the *η*_p_ value dropped to 0.714, about 20% lower than that for pure Ti. Furthermore, the *H*^3^/*E*^2^ ratio can also be used to characterize the resistance against plastic deformation [61,62]. The *H*^3^/*E*^2^ ratios of the irradiated samples were significantly higher than those of pure Ti, and increased with the larger number of IPIB pulses. This result indicates that the formation of Ti-Cr alloy coatings and the increase in IPIB irradiation pulses contribute to enhancing the resistance to plastic deformation. 

The *H*/*E* and *H*^3^/*E*^2^ ratios are widely recognized as parameters that are closely related to the tribological properties of materials [63,64], as they carry information about resistance to elastic strain to failure and resistance to plastic deformation, respectively. A hard surface can resist abrasive wear, and a lower elastic modulus can facilitate elastic deformation to absorb energy under contact stress [65]. Thus, materials with higher *H*/*E* and *H*^3^/*E*^2^ ratios generally exhibit favorable wear resistance [66,67]. In this study, the Ti-Cr alloy coating was formed after 1 pulse, which resulted in a change in the *H*/*E* ratio from 0.018 to 0.028 and the *H*^3^/*E*^2^ ratio from 0.0007 to 0.0029 (Table 1). Subsequent IPIB pulses improved the hardness and reduced the elastic modulus, leading to a further increase in the *H*/*E* and *H*^3^/*E*^2^ ratios. After 20 pulses, the Ti-Cr alloy coating sample showed *H*/*E* and *H*^3^/*E*^2^ ratios that were 2.7 and 15.9 times higher than those of pure Ti, respectively. It is suggested that the formation of Ti-Cr alloy coating via IPIBMM can enhance the wear resistance of the material compared to pure Ti, and the wear resistance of coatings can be improved by further irradiation. 

## 4. Conclusions

In this work, we investigated the microstructure evolution and mechanical properties of a metastable Ti-based gradient coating fabricated via IPIBMM. The main results are summarized as follows:

(1) The designed fabrication of Ti-Cr alloy coatings on Ti substrate was successfully achieved using the IPIBMM method. The melting depth observed in the experiment was 1.15 μm, which is in close agreement with the calculated value.
(2) The increase in the IPIB pulse number leads to more homogenized element distribution and the formation of compositional gradient layers, despite the reduction in thickness of the alloy layer due to surface sputtering. The resulting gradient Ti-Cr alloy coating is metallurgically bonded to the Ti substrate, with a thickness of ~770 nm.
(3) Microstructural analysis revealed that IPIB irradiation caused the formation of craters and cracks on the surface. However, further irradiation eliminated these defects and led to a smoother surface, primarily due to the homogenization of element distribution and the formation of a compositional gradient layer.
(4) The IPIBMM process induced the generation of metastable structures in the Ti-Cr alloy coatings. The addition of Cr facilitated the lattice transition in Ti from α-Ti to β-Ti. IPIB irradiation resulted in the formation of the supersaturated solid solution structures β-Ti (Cr) and Cr (Ti) and a change of the preferred orientation of Cr from (110) to (200).
(5) Compared with pure Ti, Ti-Cr alloying coating samples fabricated via IPIBMM displayed higher hardness, plastic factor *η*_p_, and *H*/*E* and *H*^3^/*E*^2^ ratios, which increased significantly with further irradiation. This indicates that the surface alloying of Cr by IPIBMM is an effective strategy to improve the hardness, plastic deformation resistance, and wear resistance of pure Ti.

This works sheds light on IPIBMM as a reliable and efficient method for the preparation of composition gradient alloy coatings with robust adhesion and desirable performance. The approach is extendable to various material systems and provides a promising solution for the development of composition gradient coatings in environments requiring high adhesion or performance induced by metastable structures.

## Figures and Tables

**Table 1:** Nanoindentation results of pure Ti and Ti-Cr alloy coating samples with different pulses of IPIB.

Sample	*E* (GPa)	*H* (GPa)	*η*_p_	*H*/*E*	*H*^3^/*E*^2^
original, pure Ti	2.3	125.5	0.887	0.018	0.0007
irradiated, 1 pulse	3.8	135.9	0.845	0.028	0.0029
irradiated, 5 pulses	4.2	114.6	0.803	0.036	0.0055
irradiated, 20 pulses	4.8	100.3	0.714	0.048	0.0111

## Data Availability

The data are contained within the article.
